# Regular use of fish oil supplements and course of cardiovascular diseases: prospective cohort study

**DOI:** 10.1136/bmjmed-2022-000451

**Published:** 2024-05-21

**Authors:** Ge Chen, Zhengmin (Min) Qian, Junguo Zhang, Shiyu Zhang, Zilong Zhang, Michael G Vaughn, Hannah E Aaron, Chuangshi Wang, Gregory YH Lip, Hualiang Lin

**Affiliations:** 1 Department of Epidemiology, Sun Yat-Sen University, Guangzhou, China; 2 Department of Epidemiology and Biostatistics, College for Public Health and Social Justice, Saint Louis University, Saint Louis, Missouri, USA; 3 School of Social Work, College for Public Health and Social Justice, Saint Louis University, Saint Louis, Missouri, USA; 4 Medical Research and Biometrics Centre, Fuwai Hospital, National Centre for Cardiovascular Diseases, Peking Union Medical College, Beijing, China; 5 Liverpool Centre for Cardiovascular Science, University of Liverpool and Liverpool Heart and Chest Hospital, Liverpool, UK; 6 Department of Clinical Medicine, Aalborg University, Aalborg, Denmark

**Keywords:** Health policy, Cardiology, Nutritional sciences, Public health

## Abstract

**Objective:**

To examine the effects of fish oil supplements on the clinical course of cardiovascular disease, from a healthy state to atrial fibrillation, major adverse cardiovascular events, and subsequently death.

**Design:**

Prospective cohort study.

**Setting:**

UK Biobank study, 1 January 2006 to 31 December 2010, with follow-up to 31 March 2021 (median follow-up 11.9 years).

**Participants:**

415 737 participants, aged 40-69 years, enrolled in the UK Biobank study.

**Main outcome measures:**

Incident cases of atrial fibrillation, major adverse cardiovascular events, and death, identified by linkage to hospital inpatient records and death registries. Role of fish oil supplements in different progressive stages of cardiovascular diseases, from healthy status (primary stage), to atrial fibrillation (secondary stage), major adverse cardiovascular events (tertiary stage), and death (end stage).

**Results:**

Among 415 737 participants free of cardiovascular diseases, 18 367 patients with incident atrial fibrillation, 22 636 with major adverse cardiovascular events, and 22 140 deaths during follow-up were identified. Regular use of fish oil supplements had different roles in the transitions from healthy status to atrial fibrillation, to major adverse cardiovascular events, and then to death. For people without cardiovascular disease, hazard ratios were 1.13 (95% confidence interval 1.10 to 1.17) for the transition from healthy status to atrial fibrillation and 1.05 (1.00 to 1.11) from healthy status to stroke. For participants with a diagnosis of a known cardiovascular disease, regular use of fish oil supplements was beneficial for transitions from atrial fibrillation to major adverse cardiovascular events (hazard ratio 0.92, 0.87 to 0.98), atrial fibrillation to myocardial infarction (0.85, 0.76 to 0.96), and heart failure to death (0.91, 0.84 to 0.99).

**Conclusions:**

Regular use of fish oil supplements might be a risk factor for atrial fibrillation and stroke among the general population but could be beneficial for progression of cardiovascular disease from atrial fibrillation to major adverse cardiovascular events, and from atrial fibrillation to death. Further studies are needed to determine the precise mechanisms for the development and prognosis of cardiovascular disease events with regular use of fish oil supplements.

**WHAT IS ALREADY KNOWN ON THIS TOPIC**

- Findings of the effects of omega 3 fatty acids or fish oil on the risk of cardiovascular disease are controversial
- Most previous studies focused on one health outcome and did not characterise specific cardiovascular disease outcomes (eg, atrial fibrillation, myocardial infarction, stroke, heart failure, and major adverse cardiovascular events)
- Whether fish oil could differentially affect the dynamic course of cardiovascular diseases, from atrial fibrillation to major adverse cardiovascular events, to other specific cardiovascular disease outcomes, or even to death, is unclear

**WHAT THIS STUDY ADDS**

- In people with no known cardiovascular disease, regular use of fish oil supplements was associated with an increased relative risk of atrial fibrillation and stroke
- In people with known cardiovascular disease, the beneficial effects of fish oil supplements were seen on transitions from atrial fibrillation to major adverse cardiovascular events, atrial fibrillation to myocardial infarction, and heart failure to death

**HOW THIS STUDY MIGHT AFFECT RESEARCH, PRACTICE, OR POLICY**

- Regular use of fish oil supplements might have different roles in the progression of cardiovascular disease
- Further studies are needed to determine the precise mechanisms for the development and prognosis of cardiovascular disease events with regular use of fish oil supplements

## Introduction

Cardiovascular disease is the leading cause of death worldwide, accounting for about one sixth of overall mortality in the UK.^1 2^ Fish oil, a rich source of omega 3 fatty acids, containing eicosapentaenoic acid and docosahexaenoic acid, has been recommended as a dietary measure to prevent cardiovascular disease.^3^ The UK National Institute for Health and Care Excellence recommends that people with or at high risk of cardiovascular disease consume at least one portion of oily fish a week, and the use of fish oil supplements has become popular in the UK and other western countries in recent years.^4 5^

Although some epidemiological and clinical studies have assessed the effect of omega 3 fatty acids or fish oil on cardiovascular disease and its risk factors, the findings are controversial. The Agency for Healthcare Research and Quality systematically reviewed 37 observational studies and 61 randomised controlled trials, and found evidence indicating the beneficial effects of higher consumption of fish oil supplements on ischaemic stroke, whereas no beneficial effect was found for atrial fibrillation, major adverse cardiovascular events, myocardial infarction, total stroke, or all cause death.^6^ In contrast, the Reduction of Cardiovascular Events with Icosapent Ethyl-Intervention Trial (REDUCE-IT) reported a decreased risk of major adverse cardiovascular events with icosapent ethyl in patients with raised levels of triglycerides, regardless of the use of statins.^7^ Most of these findings, however, tended to assess the role of fish oil at a certain stage of cardiovascular disease. For example, some studies restricted the study population to people with a specific cardiovascular disease or at a high risk of cardiovascular disease,^8 9^ whereas others evaluated databases of generally healthy populations.^10^ All of these factors might preclude direct comparison of the effects of omega 3 fatty acids on atrial fibrillation events or on further deterioration of cardiovascular disease. Few studies have fully characterised specific cardiovascular disease outcomes or accounted for differential effects based on the complex disease characteristics of participants. Hence, in this study, we hypothesised that fish oil supplements might have harmful, beneficial, or no effect on different cardiovascular disease events in patients with varying health conditions.

Most previous studies on the association between fish oil and cardiovascular diseases generally focused on one health outcome. Also, no study highlighted the dynamic progressive course of cardiovascular diseases, from healthy status (primary stage), to atrial fibrillation (secondary stage), major adverse cardiovascular events (tertiary stage), and death (end stage). Clarifying this complex pathway in relation to the detailed progression of cardiovascular diseases would provide substantial insights into the prevention or treatment of future disease at critical stages. Whether fish oil could differentially affect the dynamic course of cardiovascular disease (ie, from atrial fibrillation to major adverse cardiovascular events, to other specific cardiovascular disease outcomes, or even to death) is unclear.

To deal with this evidence gap, we conducted a longitudinal cohort study to estimate the associations between fish oil supplements and specific clinical cardiovascular disease outcomes, including atrial fibrillation, major adverse cardiovascular events, and all cause death in people with no known cardiovascular disease or at high risk of cardiovascular disease for the purpose of primary prevention. We also assessed the modifying effects of fish oil supplements on the disease process, from atrial fibrillation to other outcomes, in people with known cardiovascular disease for the purpose of secondary prevention.

## Methods

The UK Biobank is a community based cohort study with more than half a million UK inhabitants aged 40-69 years at recruitment.^11–13^ Participants were invited to participate in this study if they were registered with the NHS and lived within 35 km of one of 22 Biobank assessment centres. Between 1 March 2006 and 31 July 2010, a baseline survey was conducted, based on a touch screen questionnaire and face-to-face interviews, to collect detailed personal, socioeconomic, and lifestyle characteristics, and information on diseases.^11–13^

**Figure 1:**
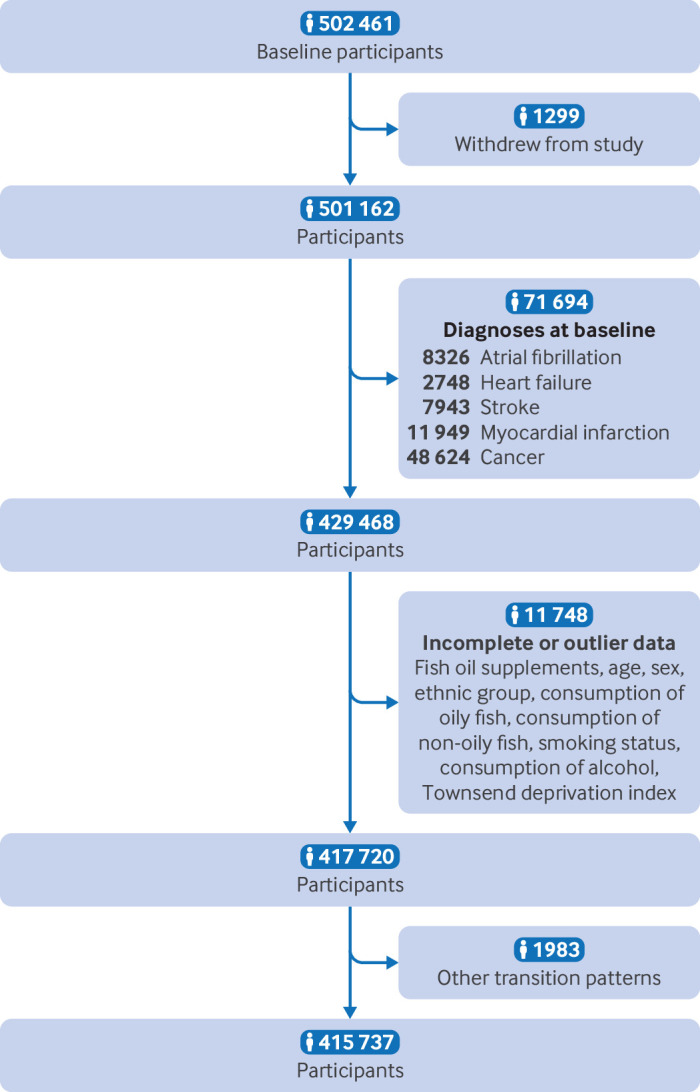
Flowchart of selection of participants in study. The count of diagnosed diseases does not equate to the total number of individuals, because each person could have multiple diagnoses

We excluded patients who had a diagnosis of atrial fibrillation (n=8326), heart failure (n=2748), myocardial infarction (n=11 949), stroke (n=7943), or cancer (n=48 624) at baseline; who withdrew from the study during follow-up (n=1299); or who had incomplete or outlier data for the main information (n=11 748). Because we focused only on a specific sequence of progression of cardiovascular disease (ie, from healthy status to atrial fibrillation, to major adverse cardiovascular events, and then to death), we excluded 1983 participants with other transition patterns. The remaining 415 737 participants were included in this analysis (figure 1).

### Determining use of fish oil supplements

Information on regular use of fish oil supplements was collected from a self-reported touchscreen questionnaire during the baseline survey.^14 15^ Each participant was asked whether they regularly used any fish oil supplement. Trained staff conducted a verbal interview with participants, asking if they were currently receiving treatments or taking any medicines, including omega 3 or fish oil supplements. Based on this information, we classified participants as regular users of fish oil supplements and non-users.

### Follow-up and outcomes

Participants were followed up from the time of recruitment to death, loss to follow-up, or the end date of follow-up (31 March 2021), whichever came first. Incident cases of interest, including atrial fibrillation, heart failure, stroke, and myocardial infarction, were identified by linkage to death registries, primary care records, and hospital inpatient records.^11^ Information on deaths was obtained from death registries of the NHS Information Centre, for participants in England and Wales, and from the NHS Central Register Scotland, for participants in Scotland.^11^ Outcomes were defined by a three character ICD-10 (international classification of diseases, 10th revision) code. In this study, atrial fibrillation was defined by ICD-10 code I48, and major adverse cardiovascular events was determined by a combination of heart failure (I50, I11.0, I13.0, and I13.2), stroke (I60-I64), and myocardial infarction (I21, I22, I23, I24.1, and I25.2) codes.

### Covariates

We collected baseline data on age (<65 years and ≥65 years), sex (men and women), ethnic group (white and non-white), Townsend deprivation index (with a higher score indicating higher levels of deprivation), smoking status (never, previous, and current smokers), and alcohol consumption (never, previous, and current drinkers). Data for sex were taken from information in UK Biobank rather than from patient reported gender. Baseline dietary data were obtained from a dietary questionnaire completed by the patient or by an interviewer. The questionnaire was established for each nation (ie, England, Scotland, and Wales) to assess an individual's usual food intake (oily fish, non-oily fish, vegetables, fruit, and red meat). Diabetes mellitus was defined by ICD-10 codes E10-E14, self-reported physician's diagnosis, self-reported use of antidiabetic drugs, or haemoglobin A1c level ≥6.5% at baseline. Hypertension was defined by ICD-10 code I10 or I15, self-reported physician's diagnosis, self-reported use of antihypertensive drugs, or measured systolic and diastolic blood pressure ≥130/85 mm Hg at baseline. Information on other comorbidities (obesity (ICD-10 code E66), chronic obstructive pulmonary disease (J44), and chronic renal failure (N18)) was extracted from the first occurrence (UKB category ID 1712). Information on the use of drugs, including antihypertensive drugs, antidiabetic drug, and statins, was extracted from treatment and drug use records. Biochemistry markers were measured immediately at the central laboratory from serum samples collected at baseline. Binge drinking was defined as consumption of ≥6 standard drinks/day for women or ≥8 standard drinks/day for men. Detailed information on alcohol consumption and binge drinking in the UK Biobank was reported previously.^16^

### Statistical analysis

Characteristics of participants are summarised as number (percentages) for categorical variables and mean (standard deviation (SD)) for continuous variables. Comparisons between regular users of fish oil supplements and non-users were made with the χ^2^ test or Student's t test.

**Figure 2:**
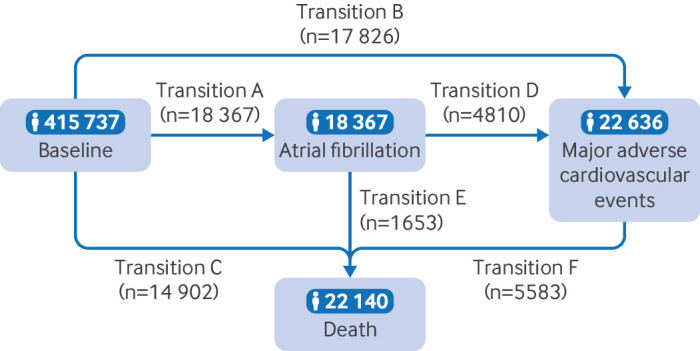
Numbers of participants in transition pattern I, from baseline to atrial fibrillation, major adverse cardiovascular events, and death

We used a multi-state regression model to assess the role of regular use of fish oil supplements in the temporal disease progression from healthy status to atrial fibrillation, to major adverse cardiovascular events, and subsequently to death. The multi-state model is an extension of competing risks survival analysis.^17–19^ The model allows simultaneous estimation of the role of risk factors in transitions from a healthy state to atrial fibrillation (transition A), healthy state to major adverse cardiovascular events (transition B), healthy state to death (transition C), atrial fibrillation to major adverse cardiovascular events (transition D), atrial fibrillation to death (transition E), and major adverse cardiovascular events to death (transition F) (transition pattern I, figure 2). The focus on these six transitions rather than on all possible health state transitions was preplanned and evidence based. If participants entered different states on the same date, we used the date of the theoretically previous state as the entry date of the latter state minus 0.5 days.

We further examined the effects of regular use of fish oil supplements on other pathways. For example, we divided major adverse cardiovascular events into three individual diseases (heart failure, stroke, and myocardial infarction), resulting in three independent pathways (transition patterns II, III, and IV, online supplemental figures S1–S3). All models were adjusted for age, sex, ethnic group, Townsend deprivation index, consumption of oily fish, consumption of non-oily fish, smoking status, alcohol consumption, obesity, hypertension, diabetes mellitus, chronic obstructive pulmonary disease, chronic renal failure, and use of statins, antidiabetic drugs, and antihypertensive drugs.

10.1136/bmjmed-2022-000451.supp2 Supplementary data

We conducted several sensitivity analyses for the multi-state analyses of transition pattern A: additionally adjusting for setting (urban and rural), body mass index (underweight, normal, overweight, and obese), and physical activity (low, moderate, and high) in the model; adjusting for binge drinking rather than alcohol consumption; additionally adjusting for other variables of dietary intake (consumption of vegetables, fruit, and red meat); calculating participants' entry date into the previous state with different time intervals (0.5 years, one year, and two years); excluding participants who entered different states on the same date; excluding events occurring in the first two years of follow-up; restricting the follow-up date to 31 March 2020 to evaluate the influence of the covid-19 pandemic; and the use of the inverse probability weighted method to deal with biases between the regular users and non-users of fish oil supplements. Also, we conducted grouped analyses for sex, age group, ethnic group, smoking status, consumption of oily fish, consumption of non-oily fish, hypertension, and drug use, to examine effect modification. The interactions were tested with the likelihood ratio test. All analyses were carried out with R software (version 4.0.3), and the multi-model analysis was performed with the mstate package. A two tailed P value <0.05 was considered significant.

### Patient and public involvement

Patients and/or the public were not involved in the design, or conduct, or reporting, or dissemination plans of this research. Participants were involved in developing the ethics and governance framework for UK Biobank and have been engaged in the progress of UK Biobank through follow-up questionnaires and additional assessment visits. UK Biobank keeps participants informed of all research output through the study website (https://www.ukbiobank.ac.uk/explore-your-participation), participant events, and newsletters.

## Results

A total of 415 737 participants (mean age 55.9 (SD 8.1) years; 55% women), aged 40-69 years, were analysed, and 31.4% (n=1 30 365) of participants reported regular use of fish oil supplements at baseline (figure 1). Table 1 shows the characteristics of regular users (n=130 365) and non-users (n=285 372) of fish oil supplements. In the group of regular users of fish oil supplements, we found higher proportions of elderly people (22.6% *v* 13.9%), white people (95.1% *v* 94.2%), and women (57.6% *v* 53.9%), and higher consumption of alcohol (93.1% *v* 92.0%), oily fish (22.1% *v* 15.4%), and non-oily fish (18.0% *v* 15.4%) than non-users. The Townsend deprivation index (mean −1.5 (SD 3.0) *v* −1.3 (3.0)) and the proportion of current smokers (8.1% *v* 11.4%) were lower in regular users of fish oil supplements. Online supplemental table S1 provides more details on patient characteristics and online supplemental table S2 compares the basic characteristics of included and excluded people.

**Table 1:** Baseline characteristics of study participants grouped by use of fish oil supplements

Characteristics	All participants (n=415 737)	Non-users of supplements (n=285 372)	Users of supplements (n=130 365)
Age (years)			
<65	346 524 (83.4)	245 679 (86.1)	100 845 (77.4)
≥65	69 213 (16.6)	39 693 (13.9)	29 520 (22.6)
Sex			
Women	228 758 (55.0)	153 715 (53.9)	75 043 (57.6)
Men	186 979 (45.0)	131 657 (46.1)	55 322 (42.4)
Ethnic group			
White	392 851 (94.5)	268 845 (94.2)	124 006 (95.1)
Non-white	22 886 (5.5)	16 527 (5.8)	6359 (4.9)
Setting*			
Rural	57 474 (14.0)	38 731 (13.7)	18 743 (14.5)
Urban	354 186 (86.0)	243 654 (86.3)	110 532 (85.5)
Body mass index*			
Underweight	2149 (0.5)	1565 (0.6)	584 (0.4)
Normal weight	137 806 (33.3)	92 895 (32.7)	44 911 (34.6)
Overweight	176 041 (42.6)	119 539 (42.1)	56 502 (43.5)
Obese	97 774 (23.6)	69 950 (24.6)	27 824 (21.4)
Consumption of oily fish (times/week)			
<2	343 096 (82.5)	241 491 (84.6)	101 605 (77.9)
≥2	72 641 (17.5)	43 881 (15.4)	28 760 (22.1)
Consumption of non-oily fish (times/week)			
<2	348 253 (83.8)	241 331 (84.6)	106 922 (82.0)
≥2	67 484 (16.2)	44 041 (15.4)	23 443 (18.0)
Consumption of vegetables* (times/week)			
<2	28 365 (6.8)	21 963 (7.7)	6402 (4.9)
2-3	119 066 (28.7)	84 866 (29.8)	34 200 (26.3)
≥4	267 926 (64.5)	178 254 (62.5)	89 672 (68.3)
Consumption of fruit* (times/week)			
<2	115 111 (27.7)	88 845 (31.2)	26 266 (20.2)
2-3	169 869 (40.9)	116 655 (40.9)	53 214 (40.8)
≥4	130 467 (31.4)	79 657 (27.9)	50 810 (39.0)
Consumption of red meat* (times/week)			
<2	61 945 (14.9)	42 846 (15.0)	19 099 (14.7)
2-3	183 952 (44.3)	122 621 (43.0)	61 331 (47.1)
≥4	169 588 (40.8)	119 712 (42.0)	49 876 (38.9)
Smoking status			
Never	233 367 (56.1)	161 564 (56.6)	71 803 (55.1)
Ever	139 168 (33.5)	91 189 (32.0)	47 979 (36.8)
Current	43 202 (10.4)	32 619 (11.4)	10 583 (8.1)
Consumption of alcohol			
Never	17 911 (4.3)	12 982 (4.5)	4929 (3.8)
Ever	13 836 (3.3)	9762 (3.4)	4074 (3.1)
Current	383 990 (92.4)	262 628 (92.0)	121 362 (93.1)
Physical activity* (%)			
Low	62 892 (18.5)	46 526 (20.0)	16 366 (15.4)
Moderate	138 203 (40.8)	95 958 (41.2)	42 245 (39.8)
High	137 878 (40.7)	90 241 (38.8)	47 637 (44.8)
Townsend deprivation index (mean, SD)	−1.4 (3.1)	−1.3 (3.0)	−1.5 (3.0)

Data are number (%) unless stated otherwise.

*These variables included missing values.

Over a median follow-up time of of 11.9 years, 18 367 participants had atrial fibrillation (transition A) and 17 826 participants had major adverse cardiovascular events (transition B); 14 902 participants died without having atrial fibrillation or major adverse cardiovascular events (transition C). Among patients with incident atrial fibrillation, 4810 developed major adverse cardiovascular events (transition D) and 1653 died (transition E). Among patients with incident major adverse cardiovascular events, 5585 died during follow-up (transition F, figure 2). In separate analyses for individual diseases (transition patterns II, III, and IV, online supplemental figures S1–S3), in patients with atrial fibrillation, 3085 developed heart failure, 1180 had a stroke, and 1415 had a myocardial infarction. During follow-up, 2436, 2088, and 2098 deaths occurred in patients with heart failure, stroke, and myocardial infarction, respectively.

### Multi-state regression results

Table 2 shows the different roles of regular use of fish oil supplements in transitions from healthy status to atrial fibrillation, to major adverse cardiovascular events, and then to death. For individuals in the primary stage (healthy status), we found that the use of fish oil supplements had a harmful effect on the transition from health to atrial fibrillation, with an adjusted hazard ratio of 1.13 (95% CI 1.10 to 1.17, transition A). The hazard ratio for transition B (from health to major adverse cardiovascular events) was 1.00 (95% CI 0.97 to 1.04) and for transition C (from health to death) was 0.98 (0.95 to 1.02).

**Table 2:** Hazard ratios (95% confidence intervals) for each transition, for different transition patterns for progressive cardiovascular disease by regular use of fish oil supplements

Transition pattern	No of events	Hazard ratio (95% CI)	P value
Transition pattern I			
Baseline→atrial fibrillation	18 367	1.13 (1.10 to 1.17)	<0.001
Baseline→major adverse cardiovascular events	17 826	1.00 (0.97 to 1.04)	0.819
Baseline→death	14 902	0.98 (0.95 to 1.02)	0.280
Atrial fibrillation→major adverse cardiovascular events	4810	0.92 (0.87 to 0.98)	0.008
Atrial fibrillation→death	1653	0.91 (0.82 to 1.01)	0.680
Major adverse cardiovascular events→death	5585	0.99 (0.94 to 1.06)	0.937
Transition pattern II			
Baseline→atrial fibrillation	18 367	1.13 (1.10 to 1.17)	<0.001
Baseline→heart failure	4552	0.92 (0.86 to 0.98)	0.011
Baseline→death	17 580	0.99 (0.96 to 1.02)	0.655
Atrial fibrillation→heart failure	3085	0.95 (0.88 to 1.02)	0.162
Atrial fibrillation→death	2124	0.89 (0.82 to 0.98)	0.016
Heart failure→death	2436	0.91 (0.84 to 0.99)	0.040
Transition pattern III			
Baseline→atrial fibrillation	18 367	1.13 (1.10 to 1.17)	<0.001
Baseline→stroke	6098	1.05 (1.00 to 1.11)	0.050
Baseline→death	17 400	0.97 (0.94 to 1.01)	0.131
Atrial fibrillation→stroke	1180	1.00 (0.89 to 1.13)	0.983
Atrial fibrillation→death	2652	0.91 (0.84 to 0.99)	0.021
Stroke→death	2088	1.04 (0.95 to 1.14)	0.347
Transition pattern IV			
Baseline→atrial fibrillation	18 367	1.13 (1.10 to 1.17)	<0.001
Baseline→myocardial infarction	9300	1.01 (0.96 to 1.05)	0.711
Baseline→death	17 378	0.98 (0.95 to 1.01)	0.235
Atrial fibrillation→myocardial infarction	1415	0.85 (0.76 to 0.96)	0.006
Atrial fibrillation→death	2664	0.88 (0.81 to 0.95)	0.002
Myocardial infarction→death	2098	1.03 (0.94 to 1.13)	0.510

Model adjusted for age, sex, ethnic group, Townsend deprivation index, consumption of oily fish, consumption of non-oily fish, smoking status, consumption of alcohol, obesity, hypertension, diabetes mellitus, chronic obstructive pulmonary disease, chronic renal failure, and use of statins, antidiabetic drugs, and antihypertensive drugs.

CI, confidence interval.

For individuals in the secondary stage (atrial fibrillation) at the beginning of the study, regular use of fish oil supplements decreased the risk of major adverse cardiovascular events (transition D, hazard ratio 0.92, 95% CI 0.87 to 0.98), and had a borderline protective effect on the transition from atrial fibrillation to death (transition E, 0.91, 0.82 to 1.01). For transition F, from major adverse cardiovascular events to death, after adjusting for covariates, the hazard ratio was 0.99 (0.94 to 1.06, transition pattern I, table 2).

We divided major adverse cardiovascular events into three individual diseases (ie, heart failure, stroke, and myocardial infarction) and found that regular use of fish oil supplements was marginally associated with an increased risk of stroke in people with a healthy cardiovascular state (hazard ratio 1.05, 95% CI 1.00 to 1.11), whereas a protective effect was found in transitions from healthy cardiovascular states to heart failure (0.92, 0.86 to 0.98). For patients with atrial fibrillation, we found that the beneficial effects of regular use of fish oil supplements were for transitions from atrial fibrillation to myocardial infarction (0.85, 0.76 to 0.96), and from atrial fibrillation to death (0.88, 0.81 to 0.95) for transition pattern IV. For patients with heart failure, we found a protective effect of regular use of fish oil supplements on the risk of mortality (0.91, 0.84 to 0.99) (transition patterns II, III, and IV, table 2).

### Stratified and sensitivity analyses

We found that age, sex, smoking, consumption of non-oily fish, prevalent hypertension, and use of statins and antihypertensive drugs modified the associations between regular use of fish oil supplements and the transition from healthy states to atrial fibrillation (online supplemental figure S4). We found that the association between regular use of fish oil supplements and risk of transition from healthy states to major adverse cardiovascular events was greater in women (hazard ratio 1.06, 95% CI 1.00 to 1.11, P value for interaction=0.005) and non-smoking participants (1.06, 1.06 to 1.11, P value for interaction=0.001) (online supplemental figure S4). The protective effect of regular use of fish oil supplements on the transition from healthy states to death was greater in men (hazard ratio 0.93, 95% CI 0.89 to 0.98, P value for interaction=0.003) and older participants (0.91, 0.86 to o 0.96, P value for interaction=0.002) (online supplemental figures S5 and S6). The results were not substantially changed in the sensitivity analyses (online supplemental table S3).

## Discussion

### Principal findings

Our study characterised the regular use of fish oil supplements on the progressive course of cardiovascular disease, from a healthy state (primary stage), to atrial fibrillation (secondary stage), major adverse cardiovascular events (tertiary stage), and death (end stage). In this prospective analysis of more than 400 000 UK adults, we found that regular use of fish oil supplements could have a differential role in the progression of cardiovascular disease. For people with a healthy cardiovascular profile, regular use of fish oil supplements, a choice of primary prevention, was associated with an increased risk of atrial fibrillation. For participants with a diagnosis of atrial fibrillation, however, regular use of fish oil supplements, as secondary prevention, had a protective effect or no effect on transitions from atrial fibrillation to major adverse cardiovascular events, atrial fibrillation to death, and major adverse cardiovascular events to death. When we divided major adverse cardiovascular events into three individual diseases (ie, heart failure, stroke, and myocardial infarction), we found associations that could suggest a mildly harmful effect between regular use of fish oil supplements and transitions from a healthy cardiovascular state to stroke, whereas potential beneficial associations were found between regular use of fish oil supplements and transitions from atrial fibrillation to myocardial infarction, atrial fibrillation to death, and heart failure to death.

### Comparison with other studies

#### Primary prevention

The cardiovascular benefits of regular use of fish oil supplements have been examined in numerous studies but the results are controversial. Extending previous reports, our study estimated the associations between regular use of fish oil supplements and specific clinical cardiovascular disease outcomes in people with no known cardiovascular disease. Our findings are in agreement with the results of several previous randomised controlled trials and meta-analyses. The Long-Term Outcomes Study to Assess Statin Residual Risk with Epanova in High Cardiovascular Risk Patients with Hypertriglyceridaemia (STRENGTH) reported that consumption of 4 g/day of marine omega 3 fatty acids was associated with a 69% higher risk of new onset atrial fibrillation in people at high risk of cardiovascular disease.^20^ A meta-analysis of seven randomised controlled trials showed that users of marine omega 3 fatty acids supplements had a higher risk of atrial fibrillation events, with a hazard ratio of 1.25 (95% CI 1.07 to 1.46, P=0.013).^21^ The Vitamin D and Omega-3 Trial (VITAL Rhythm study), a large trial of omega 3 fatty acids for the primary prevention of cardiovascular disease in adults aged ≥50 years, however, found no effects on incident atrial fibrillation, major adverse cardiovascular events, or cardiovascular disease mortality among those treated with 840 mg/day of marine omega 3 fatty acids compared with placebo.^10 22^

One possible explanation for the inconsistent results in these studies is that adverse effects might be related to dose and composition. Higher doses of omega 3 fatty acids used in previous studies might have had an important role in causing an adverse effect on atrial fibrillation.^21^ One study found that high concentrations of fish oil altered cell membrane properties and inhibited Na-K-ATPase pump activity, whereas a low concentration of fish oil minimised peroxidation potential and optimised activity.^23^ In another study, individuals with atrial fibrillation or flutter had higher percentages of total polyunsaturated fatty acids, and n-3 and n-6 polyunsaturated fatty acids, on red blood cell membranes than healthy controls.^24^

In terms of composition of omega 3 fatty acids, a recent meta-analysis showed that eicosapentaenoic acid alone can be more effective at reducing the risk of cardiovascular disease than the combined effect of eicosapentaenoic acid and docosahexaenoic acid.^25^ Similar outcomes were reported in the INSPIRE study, which showed that higher levels of docosahexaenoic acid reduced the cardiovascular benefits of eicosapentaenoic acid when given as a combination.^26^ Another possible explanation is that age, sex, ethnic group, smoking status, dietary patterns, and use of statins and antidiabetic drugs by participants might modify the effects of regular use of fish oil supplements on cardiovascular disease events. Despite these differences in risk estimates, our findings do not support the use of fish oil or omega 3 fatty acid supplements for the primary prevention of incident atrial fibrillation or other specific clinical cardiovascular disease events in generally healthy individuals. Caution might be warranted when fish oil supplements are used for primary prevention because of the uncertain cardiovascular benefits.

#### Secondary prevention

Our large scale cohort study assessed the role of regular use of fish oil supplements on the disease process, from atrial fibrillation to more serious cardiovascular disease stages, to death, in people with known cardiovascular disease. Contrary to the observations for primary prevention, we found associations that could suggest beneficial effects between regular use of fish oil supplements and most cardiovascular disease transitions. No associations were found between regular use of fish oil supplements and transitions from atrial fibrillation to death, or from major adverse cardiovascular events to death.

Consistent with our hypothesis, the Gruppo Italiano per lo Studio della Sopravvivenza nell'Infarto Miocardico (GISSI) Prevenzione study reported an association between administration of low dose prescriptions of n-3 polyunsaturated fatty acids and reduced cardiovascular events in patients with recent myocardial infarction.^27^ A meta-analysis of 16 randomised controlled trials also reported a tendency towards a greater beneficial effect for secondary prevention in patients with cardiovascular disease.^28^ Why patients with previous atrial fibrillation benefit is unclear. These findings indicate that triglyceride independent effects of omega 3 fatty acids might in part be responsible for the benefits in cardiovascular disease seen in previous trials.^29–31^ No proven biological mechanism for this explanation exists, however, and the dose and formulation of omega 3 fatty acids used in clinical practice are not known.

For the disease process, from cardiovascular disease to death, our findings are consistent with the results of secondary prevention trials of omega 3 fatty acids, which have mostly shown a weak or neutral preventive effect in all cause mortality with oil fish supplements. The GISSI heart failure trial (GISSI-HF), conducted in 6975 patients with chronic heart failure, reported that supplemental omega 3 fatty acids reduced the risk of all cause mortality by 9% (hazard ratio 0.91, 95% CI 0.833 to 0.998, P=0.041).^32^ Zelniker et al showed that omega 3 fatty acids were inversely associated with a lower incidence of sudden cardiac death in patients with non-ST segment elevation acute coronary syndrome.^33^ A meta-analysis found that use of omega 3 supplements of ≤1 capsule/day was not associated with all cause mortality, but among participants with a risk of cardiovascular disease, taking a higher dose was associated with a reduction in cardiac death and sudden death.^28^ Individuals who might benefit the most from fish oil or omega 3 fatty acid supplements are possibly more vulnerable individuals, such as those with previous cardiovascular diseases and those who can no longer live in the community. How fish oil supplements stop further deterioration of cardiovascular disease is unclear, but the theory that supplemental omega 3 fatty acids might protect the coronary artery is biologically plausible, suggesting that omega 3 fatty acids have anti-inflammatory and anti-hypertriglyceridaemia effects, contributing to a reduction in thrombosis and improvement in endothelial function.^34–41^ Nevertheless, the effects of omega 3 fatty acids vary according to an individual's previous use of statins, which might partly explain the different effects of fish oil supplements in people with and without cardiovascular disease.

Many studies of omega 3 fatty acids, including large scale clinical trials and meta-analyses, have not produced entirely consistent results.^21 25 42^ Our study mainly explored the varied potential effects of regular use of fish oil supplements on progression of cardiovascular disease, offering an initial overview of this ongoing discussion. Our findings suggest caution in the use of fish oil supplements for primary prevention because of the uncertain cardiovascular benefits and adverse effects. Further studies are needed to determine whether potential confounders modify the effects of oil fish supplements and the precise mechanisms related to the development and prognosis of cardiovascular disease events.

### Strengths and limitations of this study

The strengths of our study were the large sample size, long follow-up period, which allowed us to analyse clinically diagnosed incident diseases, and complete data on health outcomes. Another strength was our analytical strategy. The multi-state model gives less biased estimates than the conventional Cox model, and distinguished the effect of regular use of fish oil supplements on each transition in the course of cardiovascular disease.

Our study had some limitations. Firstly, as an observational study, no causal relations can be drawn from our findings. Secondly, although we adjusted for multiple covariates, residual confounding could still exist. Thirdly, information on dose and formulation of the fish oil supplements was not available in this study, so we could not evaluate potential dose dependent effects or differentiate between the effects of different fish oil formulations. Fourthly, the use of hospital inpatient data for determining atrial fibrillation events could have excluded some events triggered by acute episodes, such as surgery, trauma, and similar conditions, resulting in underestimation of the true risk because undiagnosed atrial fibrillation is a common occurrence.^43^ Fifthly, most of the participants in this study were from the white ethnic group and whether the findings can be generalised to other ethnic groups is not known. Finally, our study did not consider behavioural changes in populations with different cardiovascular profiles because of limited information, and variations in outcomes for different cardiovascular states merits further exploration.

### Conclusions

This large scale prospective study of a UK cohort suggested that regular use of fish oil supplements might have differential roles in the course of cardiovascular diseases. Regular use of fish oil supplements might be a risk factor for atrial fibrillation and stroke among the general population but could be beneficial for disease progression, from atrial fibrillation to major adverse cardiovascular events, and from atrial fibrillation to death. Further studies are needed to determine whether potential confounders modify the effects of oil fish supplements and the precise mechanisms for the development and prognosis of cardiovascular disease events.

10.1136/bmjmed-2022-000451.supp1 Supplementary data

## Data Availability

Data are available upon reasonable request. UK Biobank is an open access resource. Bona fide researchers can apply to use the UK Biobank dataset by registering and applying at http://ukbiobank.ac.uk/register-apply/.
