# Supplementary material for: Regular use of fish oil supplements and course of cardiovascular diseases: prospective cohort study
Source: BMJ Med. 2024 May 21;3(1):e000451. doi: 10.1136/bmjmed-2022-000451 (PMC11116879; doi:10.1136/bmjmed-2022-000451)
Supplement: Supplementary data [file bmjmed-2022-000451supp002.pdf]

Habitual fish oil supplementation and dynamic trajectory of cardiovascular diseases: A multi-state analysis in a nationwide cohort study

Figure S1. Numbers of participants in transition pattern II from baseline to atrial fibrillation, heart failure (HF), and death.

Note: The cases of progression from AF to stroke/MI to death have been included in the AF to death pathway. AF: atrial fibrillation; HF: heart failure; MI: myocardial infarction.

Figure S2. Numbers of participants in transition pattern III from baseline to atrial fibrillation, stroke, and death.

Note: The cases of progression from AF to HF/MI to death have been included in the AF to death pathway. AF: atrial fibrillation; HF: heart failure; MI: myocardial infarction.

Figure S3. Numbers of participants in transition pattern IV from baseline to atrial fibrillation, myocardial infarction (MI), and death.

Note: The cases of progression from AF to HF/stroke to death have been included in the AF to death pathway. AF: atrial fibrillation; HF: heart failure; MI: myocardial infarction.

Table S1. Baseline characteristics of the study participants stratified by fish oil supplementation.

Note: Data were presented as mean (SD) or n (%); COPD: chronic obstructive pulmonary disease; CRF: chronic renal failure; HDL: high-density lipoprotein; LDL: low-density lipoprotein; TC: total cholesterol; TG: triglyceride.

* These variables included missing values.

Table S2. Characteristics of the participants excluded and included in the study.

Chen G, *et al. BMJMED* 2024; 3:e000451. doi: 10.1136/bmjmed-2022-000451

Note: Data were presented as mean (SD) or n (%); BMI: body mass index; COPD: chronic obstructive pulmonary disease; CRF: chronic renal failure.

Figure S4. Subgroup analysis of associations of fish supplementation with cardiovascular disease transition (transition A-B).

Note: AF: atrial fibrillation; HF: heart failure; MI: myocardial infarction; MACE: major adverse cardiovascular events. Multivariable models were adjusted for age, sex, race, Townsend deprivation index, oily fish consumption, nonoily fish consumption, smoking status, alcohol consumption, obesity, hypertension, diabetes mellitus, chronic obstructive pulmonary disease, chronic renal failure, antilipemic drug use, and antidiabetic drug use, as appropriate.

Figure S5. Subgroup analysis of associations of fish supplementation with cardiovascular disease transition (transition C-D).

Note: AF: atrial fibrillation; HF: heart failure; MI: myocardial infarction; MACE: major adverse cardiovascular events. Multivariable models were adjusted for age, sex, race, Townsend deprivation index, oily fish consumption, nonoily fish consumption, smoking status, alcohol consumption, obesity, hypertension, diabetes mellitus, chronic obstructive pulmonary disease, chronic renal failure, antilipemic drug use, and antidiabetic drug use, as appropriate.

Figure S5. Subgroup analysis of associations of fish supplementation with cardiovascular disease transition (transition E-F).

Note: AF: atrial fibrillation; HF: heart failure; MI: myocardial infarction; MACE: major adverse cardiovascular events. Multivariable models were adjusted for age, sex, race, Townsend deprivation index, oily fish consumption, nonoily fish consumption, smoking status, alcohol consumption, obesity, hypertension, diabetes mellitus, chronic obstructive pulmonary disease, chronic renal failure, antilipemic drug use, and

antidiabetic drug use, as appropriate.

Table S3. Sensitivity analysis of associations of fish oil supplementation with transitions of cardiovascular diseases.

Note: HR: hazard ratio; CI: confidence interval; AF: atrial fibrillation; HF: heart failure; MI: myocardial infarction; MACE: major adverse cardiovascular events. Multivariable models were adjusted for age, sex, race, Townsend deprivation index, oily fish consumption, nonoily fish consumption, smoking status, alcohol consumption, obesity, hypertension, diabetes mellitus, chronic obstructive pulmonary disease, chronic renal failure, antilipemic drug use, and antidiabetic drug use. as appropriate.

Chen G, *et al. BMJMED* 2024; 3:e000451. doi: 10.1136/bmjmed-2022-000451

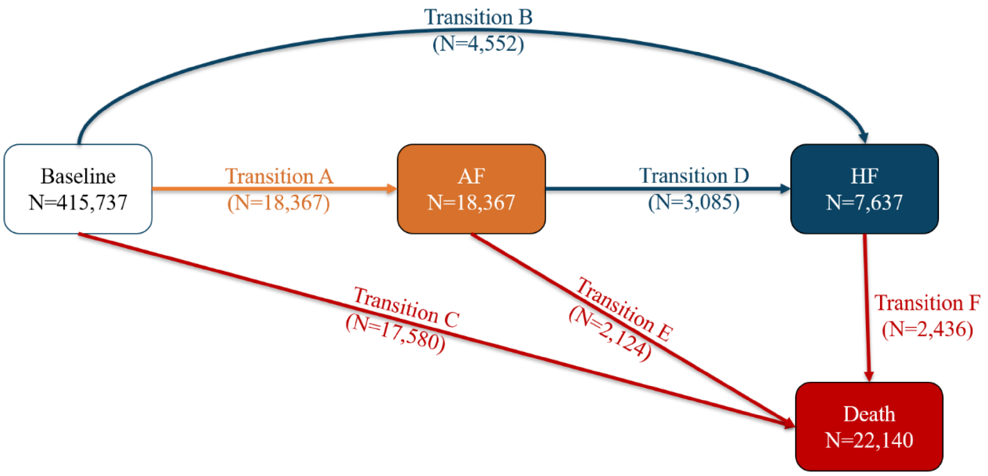

Figure S1. Numbers of participants in transition pattern II from baseline to atrial fibrillation, heart failure (HF), and death.
Note: The cases of progression from AF to stroke/MI to death have been included in the AF to death pathway. AF: atrial fibrillation; HF: heart failure; MI: myocardial infarction.

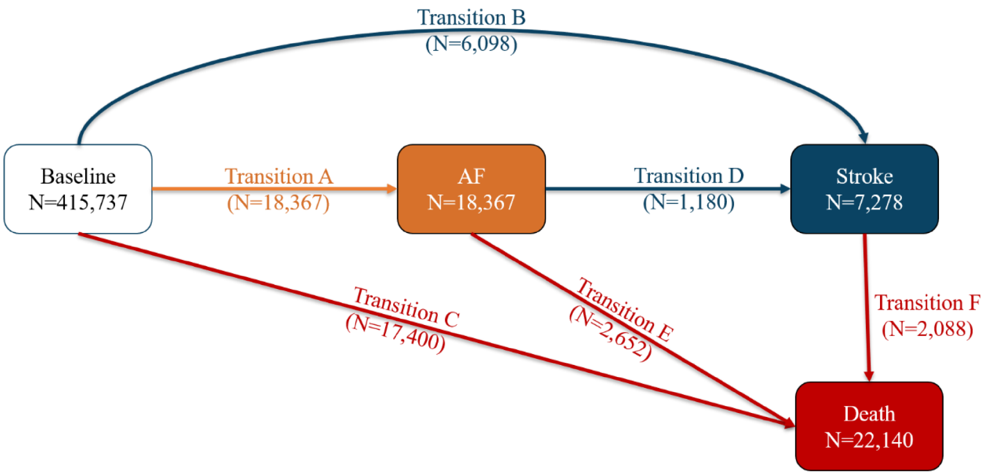

Figure S2. Numbers of participants in transition pattern III from baseline to atrial fibrillation, stroke, and death.
Note: The cases of progression from AF to HF/MI to death have been included in the AF to death pathway. AF: atrial fibrillation; HF: heart failure; MI: myocardial infarction.

Chen G, *et al. BMJMED* 2024; 3:e000451. doi: 10.1136/bmjmed-2022-000451

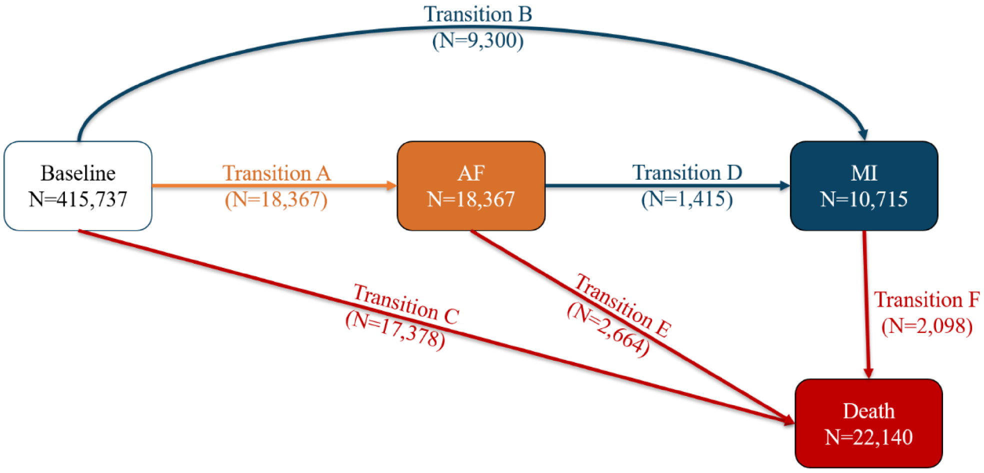

Figure S3. Numbers of participants in transition pattern IV from baseline to atrial fibrillation, myocardial infarction (MI), and death.

Note: The cases of progression from AF to HF/stroke to death have been included in the AF to death pathway. AF: atrial fibrillation; HF: heart failure; MI: myocardial infarction.

Chen G, *et al. BMJMED* 2024; 3:e000451. doi: 10.1136/bmjmed-2022-000451

Table S1. Baseline characteristics of the study participants stratified by fish oil supplementation.

Characteristics	Overall (N=415,737)	Fish oil non-users (N=285,372)	Fish oil users (N=130,365)
**History of obesity (%)**			
No	405566 (97.6)	278098 (97.5)	127468 (97.8)
Yes	10171 (2.4)	7274 (2.5)	2897 (2.2)
**History of diabetes (%)**			
No	393792 (94.7)	269761 (94.5)	124031 (95.1)
Yes	21945 (5.3)	15611 (5.5)	6334 (4.9)
**History of hypertension (%)**			
No	132971 (32.0)	95129 (33.3)	37842 (29.0)
Yes	282766 (68.0)	190243 (66.7)	92523 (71.0)
**History of COPD (%)**			
No	412710 (99.3)	283180 (99.2)	129530 (99.4)
Yes	3027 (0.7)	2192 (0.8)	835 (0.6)
**History of CRF (%)**			
No	411762 (99.0)	282687 (99.1)	129075 (99.0)
Yes	3975 (1.0)	2685 (0.9)	1290 (1.0)
**Statin use (%)**			
No	360277 (86.7)	249290 (87.4)	110987 (85.1)
Yes	55460 (13.3)	36082 (12.6)	19378 (14.9)
**Antidiabetic drug use (%)**			
No	402278 (96.8)	275638 (96.6)	126640 (97.1)
Yes	13459 (3.2)	9734 (3.4)	3725 (2.9)
**Antihypertensive drug use (%)**			
No	336183 (80.9)	232927 (81.6)	103256 (79.2)
Yes	79554 (19.1)	52445 (18.4)	27109 (20.8)
**TC* (mmol/L, mean±SD)**	5.7 ± 1.1	5.7 ± 1.1	5.8 ± 1.1
**TG* (mmol/L, mean±SD)**	1.7 ± 1.0	1.7 ± 1.0	1.7 ± 1.0
**HDL* (mmol/L, mean±SD)**	1.5 ± 0.4	1.4 ± 0.4	1.5 ± 0.4
**LDL* (mmol/L, mean±SD)**	3.6 ± 0.9	3.6 ± 0.9	3.6 ± 0.9
**HbA1c* (mmol/mol, mean±SD)**	35.9 ± 6.4	35.8 ± 6.7	35.9 ± 5.9

Note: Data were presented as mean (SD) or n (%); COPD: chronic obstructive pulmonary disease; CRF: chronic renal failure; HDL: high-density lipoprotein; LDL: low-density lipoprotein; TC: total cholesterol; TG: triglyceride.

* These variables included missing values.

Chen G, *et al. BMJMED* 2024; 3:e000451. doi: 10.1136/bmjmed-2022-000451

Table S2. Characteristics of the participants excluded and included in the study.

Characteristics	Exclude (N=86,724)	Include (N=415,737)	*P*
**Age (years, %)**			<0.001
<65	59933 (69.1)	346524 (83.4)	
≥65	26791 (30.9)	69213 (16.6)	
**Sex (%)**			<0.001
Female	44595 (51.4)	228758 (55.0)	
Male	42128 (48.6)	186979 (45.0)	
**Race (%)**			<0.001
White	79861 (95.1)	392851 (94.5)	
Non-white	4146 (4.9)	22886 (5.5)	
**Setting* (%)**			<0.001
Rural	11169 (13.0)	57474 (14.0)	
Urban	74563 (87.0)	354186 (86.0)	
**BMI* (%)**			<0.001
Underweight	477 (0.6)	2149 (0.5)	
Normal	24588 (28.7)	137806 (33.3)	
Overweight	36057 (42.1)	176041 (42.5)	
Obese	24464 (28.6)	97774 (23.6)	
**Oily fish consumption (times/week, %)**			<0.001
<2	65088 (78.8)	343096 (82.5)	
≥2	17556 (21.2)	72641 (17.5)	
**Non-oily fish consumption (times/week, %)**			<0.001
<2	68432 (82.4)	348253 (83.8)	
≥2	14569 (17.6)	67484 (16.2)	
**Vegetable consumption* (times/week, %)**			<0.001
<2	6928 (8.1)	28365 (6.8)	
2-	23090 (27.0)	119066 (28.7)	
⩾4	55402 (64.9)	267926 (64.5)	
**Fruit consumption* (times/week, %)**			<0.001
<2	23763 (27.8)	115111 (27.7)	
2-	34019 (39.8)	169869 (40.9)	
⩾4	27669 (32.4)	130467 (31.4)	
**Red meat consumption* (times/week, %)**			0.0119
<2	12688 (14.9)	61945 (14.9)	
2-	37388 (43.8)	183952 (44.3)	
⩾4	35308 (41.4)	169588 (40.8)	
**Smoking status (%)**			<0.001
Never	40130 (47.9)	233367 (56.1)	
Ever	33876 (40.4)	139168 (33.5)	
Current	9769 (11.7)	43202 (10.4)	
**Alcohol consumption (%)**			<0.001
Never	4473 (5.3)	17911 (4.3)	
Ever	4263 (5.0)	13836 (3.3)	
Current	76335 (89.7)	383990 (92.4)	
**Physical activity* (%)**			<0.001
Low	13314 (21.0)	62892 (18.6)	
Moderate	25809 (40.7)	138203 (40.8)	
High	24253 (38.3)	137878 (40.7)	
**Townsend deprive index (mean±SD)**	-1.0±3.2	-1.4±3.1	<0.001
**History of obesity (%)**			<0.001
No	83344 (96.1)	405566 (97.6)	
Yes	3380 (3.9)	10171 (2.4)	
**History of diabetes (%)**			<0.001

No	77959 (89.9)	393792 (94.7)	
Yes	8764 (10.1)	21945 (5.3)	
**History of hypertension (%)**			<0.001
No	19936 (23.0)	132971 (32.0)	
Yes	66787 (77.0)	282766 (68.0)	
**History of COPD (%)**			<0.001
No	84870 (97.9)	412710 (99.3)	
Yes	1854 (2.1)	3027 (0.7)	
**History of CRF (%)**			<0.001
No	84649 (97.6)	411762 (99.0)	
Yes	2075 (2.4)	3975 (1.0)	
**Statin use (%)**			<0.001
No	59606 (68.7)	360277 (86.7)	
Yes	27118 (31.3)	55460 (13.3)	
**Antidiabetic use (%)**			<0.001
No	81175 (93.6)	402278 (96.8)	
Yes	5549 (6.4)	13459 (3.2)	
**Antihypertensive drug use (%)**			<0.001
No	52445 (60.5)	336183 (80.9)	
Yes	34278 (39.5)	79554 (19.1)	
**TC (mmol/L, (mean±SD))**	5.5±1.2	5.7±1.1	<0.001
**TG (mmol/L, (mean±SD))**	1.8±1.0	1.7±1.0	<0.001
**HDL (mmol/L, (mean±SD))**	1.4±0.4	1.5±0.4	<0.001
**LDL (mmol/L, (mean±SD))**	3.4±0.9	3.6±0.9	<0.001
**HbA1c (mmol/mol, (mean±SD))**	37.6±8.1	35.9±6.4	<0.001

Note: Data were presented as mean (SD) or n (%); BMI: body mass index; COPD: chronic obstructive pulmonary disease; CRF: chronic renal failure; HDL: high-density lipoprotein; LDL: low-density lipoprotein; TC: total cholesterol; TG: triglyceride.

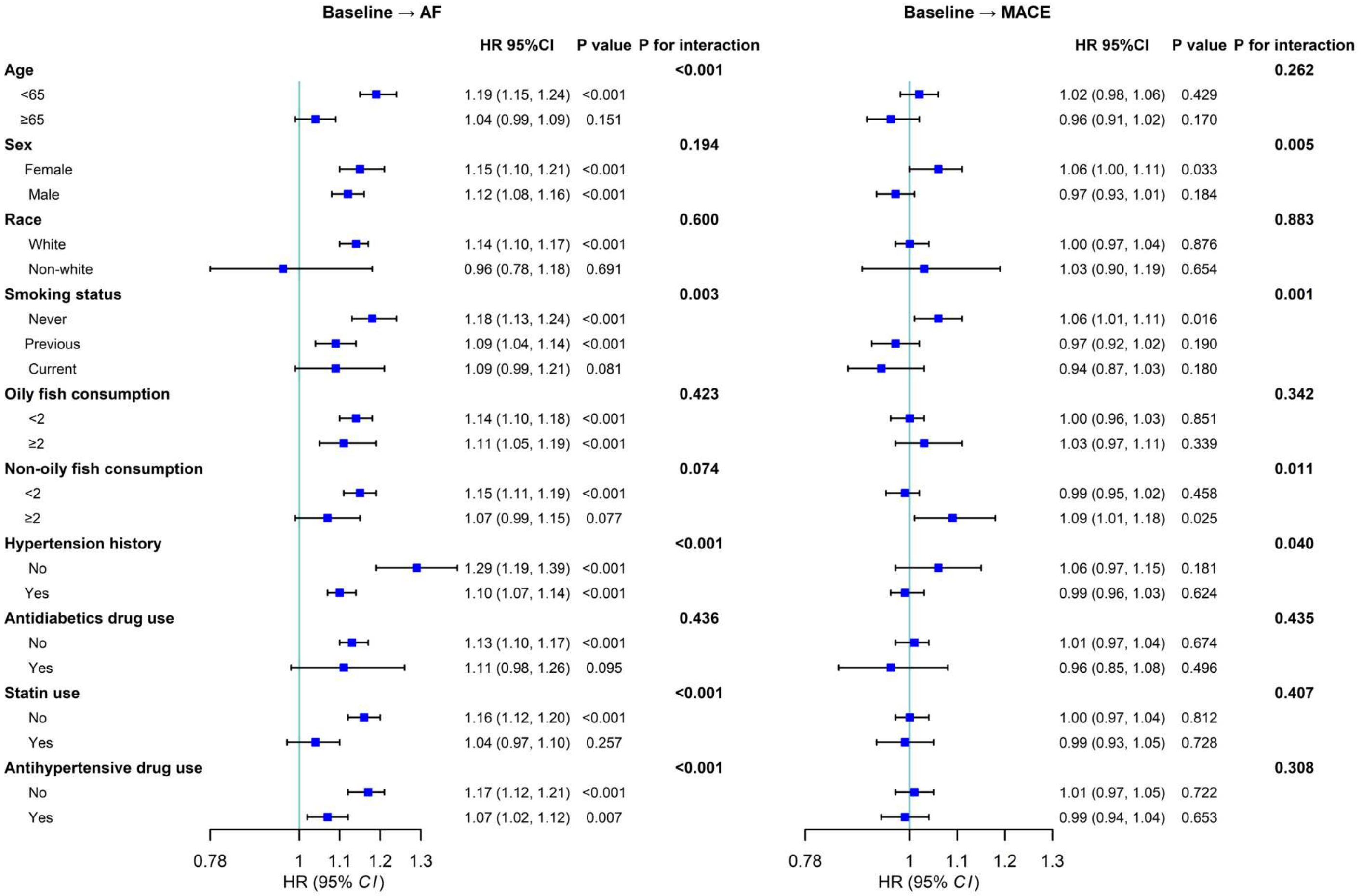

Figure S4. Subgroup analysis of associations of fish supplementation with cardiovascular disease transition (transition A-B).

Note: AF: atrial fibrillation; HF: heart failure; MI: myocardial infarction; MACE: major adverse cardiovascular events. Multivariable models were adjusted for age, sex, race, Townsend deprivation index, oily fish consumption, nonoily fish consumption, smoking status, alcohol consumption, obesity, hypertension, diabetes mellitus, chronic obstructive pulmonary disease, chronic renal failure, statin use, antidiabetic drug use and antihypertensive drug use as appropriate.

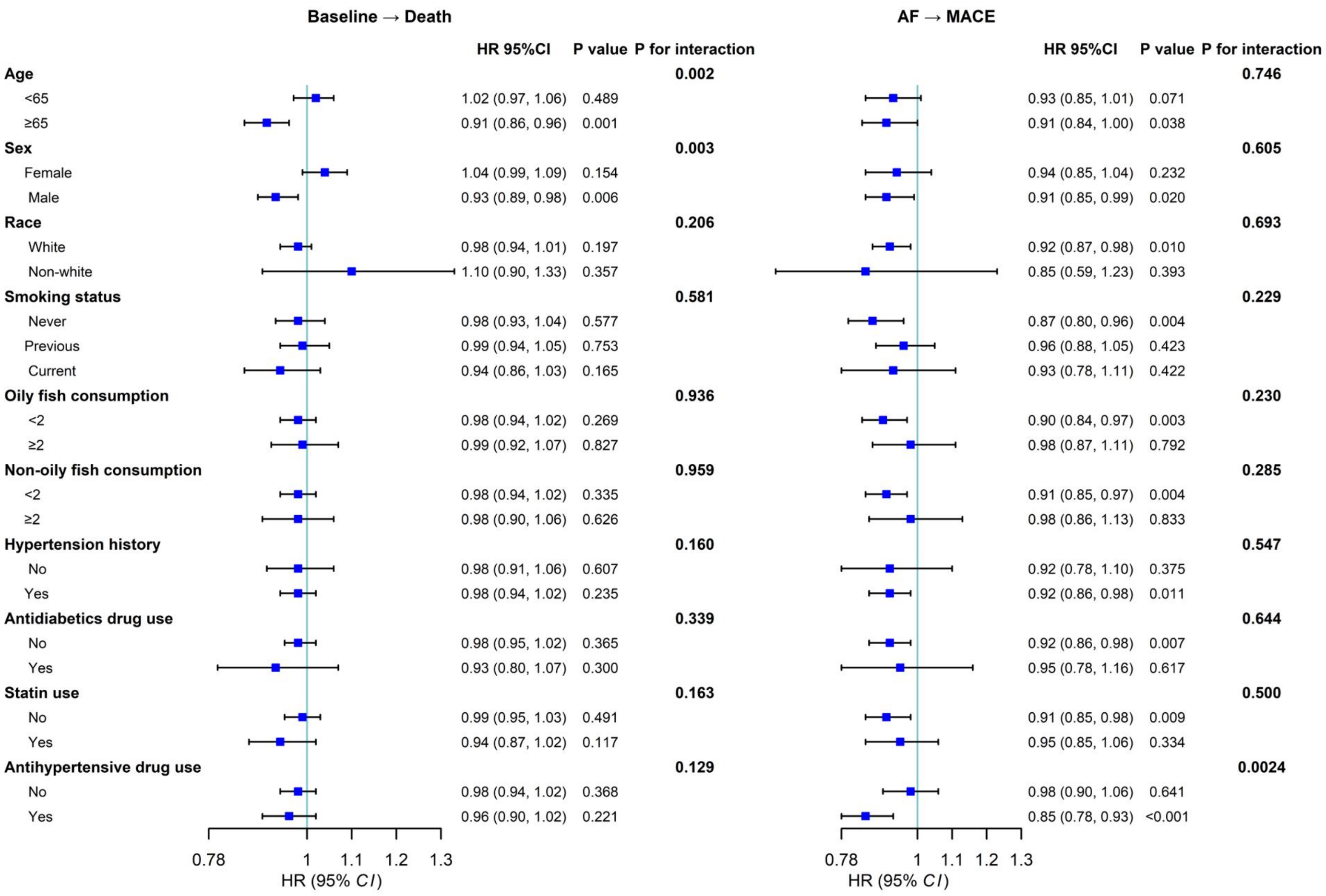

Figure S5. Subgroup analysis of associations of fish supplementation with cardiovascular disease transition (transition C-D).

BMJ Publishing Group Limited (BMJ) disclaims all liability and responsibility arising from any reliance placed on this supplemental material which has been supplied by the author(s)

Note: AF: atrial fibrillation; HF: heart failure; MI: myocardial infarction; MACE: major adverse cardiovascular events. Multivariable models were adjusted for age, sex, race, Townsend deprivation index, oily fish consumption, nonoily fish consumption, smoking status, alcohol consumption, obesity, hypertension, diabetes mellitus, chronic obstructive pulmonary disease, chronic renal failure, statin use, antidiabetic drug use and antihypertensive drug use as appropriate.

Chen G, *et al. BMJMED* 2024; 3:e000451. doi: 10.1136/bmjmed-2022-000451

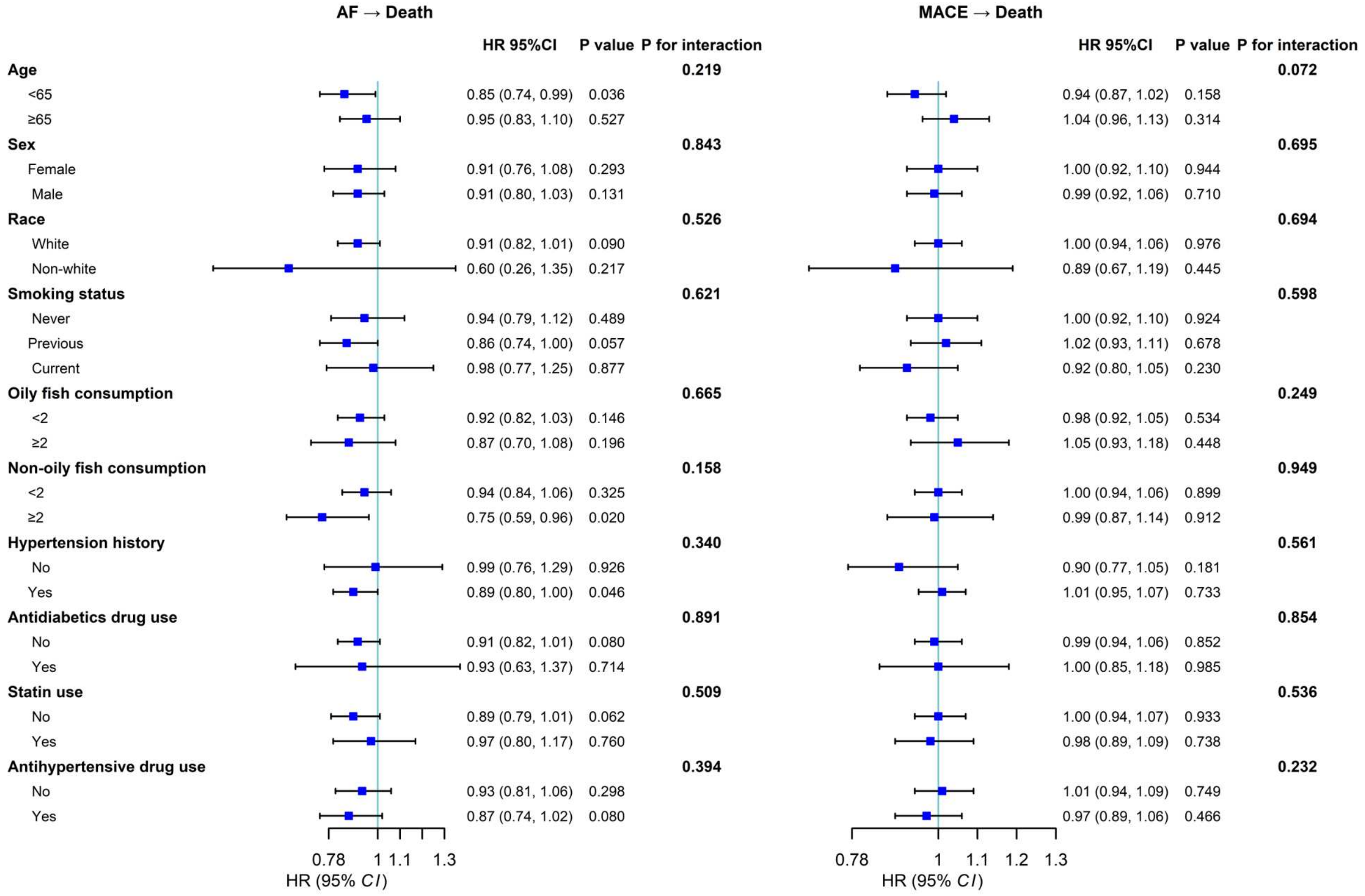

Figure S6. Subgroup analysis of associations of fish supplementation with cardiovascular disease transition (transition E-F).

BMJ Publishing Group Limited (BMJ) disclaims all liability and responsibility arising from any reliance placed on this supplemental material which has been supplied by the author(s)

Note: AF: atrial fibrillation; HF: heart failure; MI: myocardial infarction; MACE: major adverse cardiovascular events. Multivariable models were adjusted for age, sex, race, Townsend deprivation index, oily fish consumption, nonoily fish consumption, smoking status, alcohol consumption, obesity, hypertension, diabetes mellitus, chronic obstructive pulmonary disease, chronic renal failure, statin use, antidiabetic drug use and antihypertensive drug use as appropriate.

Chen G, *et al. BMJMED* 2024; 3:e000451. doi: 10.1136/bmjmed-2022-000451

Table S3. Sensitivity analysis of associations of fish oil supplementation with transitions of cardiovascular diseases.

	HR (95% *CI*)					
	Baseline → AF	Baseline → MACE	Baseline → Death	AF → MACE	AF → Death	MACE → Death
Additional adjustment for setting, body mass index and physical activity	1.17 (1.13, 1.21)*	1.03 (0.99, 1.07)	0.99 (0.96, 1.04)	0.94 (0.88, 1.00)	0.90 (0.80, 1.01)	0.97 (0.91, 1.04)
Adjustment for binge drink instead of alcohol consumption	1.13 (1.10, 1.17) *	1.01 (0.97, 1.04)	0.98 (0.95, 1.02)	0.93 (0.87, 0.99) *	0.89 (0.80, 0.99) *	0.98 (0.92, 1.04)
Additional adjustment for vegetable, fruit, and red meat consumption	1.13 (1.10, 1.16) *	1.02 (0.98, 1.05)	0.99 (0.96, 1.03)	0.94 (0.88, 0.99) *	0.93 (0.84, 1.03)	1.00 (0.95, 1.07)
Different interval						
0.5 years	1.13 (1.10, 1.17) *	1.00 (0.97, 1.04)	0.98 (0.95, 1.02)	0.92 (0.87, 0.98) *	0.91 (0.82, 1.01)	0.99 (0.94, 1.06)
1 year	1.13 (1.10, 1.17) *	1.00 (0.97, 1.04)	0.98 (0.95, 1.02)	0.92 (0.87, 0.98) *	0.91 (0.82, 1.01)	0.99 (0.94, 1.06)
2 years	1.13 (1.10, 1.17) *	1.00 (0.97, 1.04)	0.98 (0.95, 1.02)	0.93 (0.87, 0.98) *	0.91 (0.82, 1.01)	0.99 (0.94, 1.06)
Excluding participants who entered different states on the same date	1.14 (1.10, 1.18) *	1.01 (0.97, 1.04)	0.98 (0.95, 1.02)	0.91 (0.84, 0.98) *	0.91 (0.82, 1.01)	0.98 (0.92, 1.06)
Excluding events occurred in the first two-year of follow-up	1.14 (1.10, 1.17) *	1.01 (0.97, 1.04)	0.98 (0.95, 1.02)	0.91 (0.84, 0.98) *	0.91 (0.82, 1.01)	0.99 (0.92, 1.06)
Restricting the follow-up date to March 2020	1.13 (1.10, 1.17) *	1.00 (0.96, 1.03)	0.99 (0.95, 1.03)	0.93 (0.87, 0.99) *	0.88 (0.78, 0.98) *	1.02 (0.95, 1.08)
Inverse probability weighted method						
The average treatment effect	1.13 (1.10, 1.17) *	1.00 (0.97, 1.03)	0.98 (0.95, 1.02)	0.89 (0.83, 0.95) *	0.87 (0.78, 0.97) *	0.95 (0.89, 1.02)
The average treatment effect of the treated	1.11 (1.08, 1.14) *	1.00 (0.96, 1.03)	0.97 (0.94, 1.01)	0.89 (0.83, 0.96) *	0.87 (0.78, 0.97) *	0.97 (0.91, 1.03)
The average treatment effect on the controls	1.14 (1.11, 1.18) *	1.00 (0.97, 1.03)	0.98 (0.95, 1.02)	0.88 (0.82, 0.95) *	0.87 (0.78, 0.97) *	0.95 (0.89, 1.01)

Note: HR: hazard ratio; CI: confidence interval; AF: atrial fibrillation; HF: heart failure; MI: myocardial infarction; MACE: major adverse cardiovascular events. Multivariable models were adjusted for age, sex, race, Townsend deprivation index, oily fish consumption, nonoily fish consumption, smoking status, alcohol consumption, obesity, hypertension, diabetes mellitus, chronic obstructive pulmonary disease, chronic renal failure, statin use, antidiabetic drug use and antihypertensive drug use as appropriate.

* $P < 0.05$.

Chen G, *et al. BMJMED* 2024; 3:e000451. doi: 10.1136/bmjmed-2022-000451
